# Approximate Evolution for A Hybrid System—An Optomechanical Jaynes-Cummings Model

**DOI:** 10.3390/e22121373

**Published:** 2020-12-05

**Authors:** Luis Medina-Dozal, Irán Ramos-Prieto, José Récamier

**Affiliations:** Instituto de Ciencias Físicas, Universidad Nacional Autónoma de México, Apdo. Postal 48-3, Cuernavaca, Morelos 62251, Mexico; luis.medina@icf.unam.mx (L.M.-D.); iranrp123@gmail.com (I.R.-P.)

**Keywords:** Jaynes-Cummings model, hybird system evolution, Wei-Norman theorem

## Abstract

In this work, we start from a phenomenological Hamiltonian built from two known systems: the Hamiltonian of a pumped optomechanical system and the Jaynes-Cummings Hamiltonian. Using algebraic techniques we construct an approximate time evolution operator $\hat{U}\left(t\right)$ for the forced optomechanical system (as a product of exponentials) and take the JC Hamiltonian as an interaction. We transform the later with $\hat{U}\left(t\right)$ to obtain a generalized interaction picture Hamiltonian which can be linearized and whose time evolution operator is written in a product form. The analytic results are compared with purely numerical calculations using the full Hamiltonian and the agreement between them is remarkable.

## 1. Introduction

Combining two or more physical systems in the theoretical framework of hybrid quantum systems has provided novel quantum technologies. Individually, quantum systems like qubits, trapped ions, quantum harmonic oscillators, or, for example, the Jaynes-Cummings model (JCM), offer a wide range of applications on their own, to name a few. In fact, the JCM, although it is not conceived as a hybrid model, contains two systems that define the radiation-matter interaction in its simplest form [1,2,3]. This model has been the subject of extensive theoretical and experimental research due to its intrinsic relevance in quantum optics, the JCM has been generalized in several forms, for example, assuming that the interaction between the atom and the field is nonlinear in the field variables [4,5,6,7,8,9,10], incorporating a group of two-level atoms interacting with the field, the so-called Tavis-Cummings model [11], or incorporating simultaneously a nonlinear coupling between the atom and the field and a nonlinear Kerr-like medium [12,13,14]. On the other hand, one of the ingredients of the JCM, the two-level system (TLS) has been realized in superconducting (SC) circuits that offer scalability and flexibility, but a short coherence time, while atoms or spins have rather long coherence times, a consequence of weak coupling to external fields and limited scalability. This has generated an ingenious idea, which takes advantage of each of the systems separately to combine them in hybrid models [15,16,17].

Another system that is close to the conception of a hybrid system is a optomechanical cavity. This system offers a route to determine and control the quantum state of macroscopic objects. Quantum optomechanics provides motion and force detection near the fundamental limit imposed by quantum mechanics [18,19,20]. The most conventional optical cavity is the Fabry-Perot cavity, where one end mirror is fixed and the other is harmonically bound and allowed to oscillate under the action of radiation pressure from the intracavity light field of frequency $\omega_{L}$. Interest has arisen in recent decades in the motion of mechanical oscillators coupled to oscillation modes in a cavity [21,22,23], some recent applications of this type of resonators include—the LIGO project that uses gravitational wave interferometers whose optical path is modified by radiation pressure [24], the cooling of mechanical resonators for the study of the transition between quantum and classical behavior [25,26] and the amplification and measurement of nanometric scale forces [27,28]. The strong coupling regime provides a quantum interface allowing the coherent transfer of quantum states between the mechanical oscillator and the atoms. Controlled storage of quantum information will require electromagnetically induced transparency (EIT). This technique is widely used to control the absorption of weak light pulses or single photons in atomic ensembles and high finesse cavities. The EIT from a single atom in free space was reported in Reference [29]. There, the authors observed the direct extinction of a weak probe field and electromagnetically induced transparency from a single Barium ion.

About ten years ago, atom-photon interfaces were proposed as essential building blocks in quantum networks [30,31]. Here, photons are adopted as messengers due to their robustness in preserving quantum information during propagation, while atoms are suited to store the information in stationary nodes. The efficient transfer of quantum information between atoms and photons is essential and requires controlled photon absorption and emission with a very high probability.

Furthermore, although the coupling of an oscillating mirror to an atom or ion is not done directly, this can be made possible by a common coupling by adding the mode of a cavity [19,32]. In this sense, in Reference [33] the authors studied the transmission of a probe field through a hybrid optomechanical system consisting of a cavity and a mechanical oscillator with a TLS, and the strong coupling regime of a mechanical oscillator and a single atom [34]. At this point, the individual evolution of each subsystem offers a possibility of obtaining, under certain conditions, the exact evolution of the hybrid system, that is, if we know the evolution operator of each hybrid component, using the Wie-Norman theorem [35] it is possible to obtain, on the one hand, the exact or approximate solution of the dynamics of the physical observables, and on the other, the Wigner function that allows elucidating the quantum behavior of the system.

In this work we consider a hybrid optomechanical system composed of a cavity, a mechanical oscillator and a two level atom inside the cavity. The cavity mode is coupled to a TLS and together they are coupled to the vibrational modes of the mirror. Moreover, the optomechanical cavity is pumped by an external laser of frequency $\omega_{L}$ and amplitude Ω. Consequently, since we know the evolution operators of both subsystems, the JCM and optomechanical cavity, we construct an approximate time evolution operator for the hybrid system and evaluate the temporal evolution of several observables like the number of photons, and phonons. The paper is organized as follows: In Section 2 we present the basic theory to obtain a time evolution operator for a tripartite system composed of a forced optomechanical Hamiltonian a one mode cavity and a two level atom inside the cavity. In Section 3 we write the observables of interest in a generalized interaction picture and in Section 4 we present our numerical results and conclusions.

## 2. Theory

We begin by considering a hybrid optomechanical system whose Hamiltonian is

(1) $$\frac{\hat{H}}{\hbar }=\frac{{\hat{H}}_{0}}{\hbar }+\frac{\omega_{a}}{2}{\hat{\sigma }}_{z}+\lambda \left(\hat{a}{\hat{\sigma }}_{+}+{\hat{a}}^{†}{\hat{\sigma }}_{-}\right),$$

with $\hbar \omega_{a}$ the energy difference between the ground and the excited atomic states, λ the coupling constant between the field and the two level atom and ${\hat{H}}_{0}$ describing the simplest pumped optomechanical system given by [36,37,38,39]

(2) $$\frac{{\hat{H}}_{0}}{\hbar }=\frac{{\hat{H}}_{o}}{\hbar }+\Omega \cos\left(\omega_{L}t\right)\left(\hat{a}+{\hat{a}}^{†}\right),$$

where

(3) $$\frac{{\hat{H}}_{o}}{\hbar }=\omega_{c}\hat{n}+\omega_{m}\hat{N}-G\hat{n}\left(\hat{b}+{\hat{b}}^{†}\right).$$

Here $\omega_{c}$, $\omega_{m}$ are the field and the mechanical oscillator frequencies, $\hat{n}={\hat{a}}^{†}\hat{a}$, $\hat{N}={\hat{b}}^{†}\hat{b}$ are the number operators for the field and the mechanical oscillator and *G* is the coupling constant between the field and the mechanical oscillator given by: $G=\frac{\omega_{c}}{L}{\left(\hbar /2m\omega_{m}\right)}^{1/2}$, with *L* the equilibrium cavity length, Ω is related to the input laser power, $\omega_{L}$ is the frequency of the driving field, $\hat{a}$, (${\hat{a}}^{†}$) are the annihilation (creation) field operators. The Hamiltonian given by (1), has already been used in models where *hybridization* plays a major role, for example: division of the optical and mechanical fluctuation spectra [40], photon blockade and antibunching [33,41] and in state transfer and entanglement in trapped ions [32].

We have developed a useful approach to find an approximate time evolution operator for the Hamiltonian ${\hat{H}}_{0}$ when the system does not interact with the environment [42]. Here we use a similar approach to obtain the time evolution operator of the hybrid system described by the Hamiltonian given in (1). The first thing to take into account is that the time evolution operator associated with ${\hat{H}}_{0}$ is given by:

(4) $$\begin{array}{cc} {\hat{U}}_{0}\left(t\right) & =e^{\delta +\frac{1}{2}{\left|\beta \right|}^{2}}e^{\alpha_{1}\hat{n}}e^{\alpha_{2}\hat{N}}e^{(\alpha_{3}+|\alpha_{4}{|}^{2}/2){\hat{n}}^{2}}{\hat{D}}_{\hat{b}}\left(\alpha_{4}\hat{n}\right){\hat{D}}_{\hat{a}}\left(\beta \right), \end{array}$$

where ${\hat{D}}_{\hat{A}}\left(\alpha \right)=e^{\alpha {\hat{A}}^{†}-\alpha^{*}\hat{A}}$ is the Glauber displacement operator. This expression for the time evolution operator is valid whenever $G/\omega_{m}\ll 1$ a condition usually satisfied in the quantum optical regime and used in several experimental systems, see for instance Reference [19]. The functions $\alpha_{i}$, β and δ are given explicitly by

(5) $$\begin{array}{cc} \alpha_{1} & =-i\omega_{c}t,\\ \alpha_{2} & =-i\omega_{m}t,\\ \alpha_{3} & =-{\left(\frac{G}{\omega_{m}}\right)}^{2}\left[-i\omega_{m}t+\left(1-e^{-i\omega_{m}t}\right)\right],\\ \alpha_{4} & =-\frac{G}{\omega_{m}}\left(1-e^{i\omega_{m}t}\right). \end{array}$$

and

(6) $$\begin{array}{cc} \dot{\beta } & =-i\Omega \cos\left(\omega_{L}t\right)e^{i\omega_{c}t},\\ \dot{\gamma } & =-i\Omega \cos\left(\omega_{L}t\right)e^{-i\omega_{c}t},\\ \dot{\delta } & =\beta \dot{\gamma }, \end{array}$$

as can be seen in Equations (A2) and (A9) in the Appendix A.

Here, it is important to note that in contrast to the Fock states, the coherent states are the quantum states whose statistical behavior most resemble the classical one, this has generated considerable interest in using micro-mirrors for the generation of coherent mechanical states or even superposition of them if such micro-mirrors can be cooled to their quantum ground states [43,44,45,46,47,48].

Once we have obtained the time evolution operator corresponding to the Hamiltonian ${\hat{H}}_{0}$ we transform the interaction to get the approximate interaction Hamiltonian

(7) $${\hat{H}}_{I}^{\left(1\right)}=\frac{\hbar \omega_{a}}{2}{\hat{\sigma }}_{z}+\hbar \lambda \left[\left(\hat{a}+\beta \right){\hat{\sigma }}_{+}e^{-i\omega_{c}t}+\left({\hat{a}}^{†}+\beta^{*}\right){\hat{\sigma }}_{-}e^{i\omega_{c}t}\right],$$

where we have maintained the same level of approximation as the one used to get Equation (A7) that is, we neglect terms proportional to $G/\omega_{m}$ and ${\left(G/\omega_{m}\right)}^{2}$ as compared to one. The time evolution operator in the interaction picture satisfies the equation

(8) $$i\hbar \frac{\partial {\hat{U}}_{I}^{\left(1\right)}}{\partial t}={\hat{H}}_{I}^{\left(1\right)}{\hat{U}}_{I}^{\left(1\right)},\;\;\;{\hat{U}}_{I}^{\left(1\right)}\left(0\right)=1.$$

It is convenient now to write the interaction Hamiltonian as a the sum of a Hamiltonian containing the set $\left\{{\hat{\sigma }}_{+},{\hat{\sigma }}_{-},{\hat{\sigma }}_{z}\right\}$ and another with $\left\{\hat{a}{\hat{\sigma }}_{+},{\hat{a}}^{†}{\hat{\sigma }}_{-}\right\}$.

(9) $${\hat{H}}_{I}^{\left(1\right)}={\hat{H}}_{1}^{\left(1\right)}+{\hat{H}}_{2}^{\left(1\right)},\;\;\;\;{\hat{U}}_{I}^{\left(1\right)}={\hat{U}}_{1}^{\left(1\right)}{\hat{U}}_{2}^{\left(1\right)},$$

with

(10) $$\begin{array}{cc} {\hat{H}}_{1}^{\left(1\right)} & =\frac{\hbar \omega_{a}}{2}{\hat{\sigma }}_{z}+\hbar \lambda \left(\beta e^{-i\omega_{c}t}{\hat{\sigma }}_{+}+\beta^{*}e^{i\omega_{c}t}{\hat{\sigma }}_{-}\right),\\ {\hat{H}}_{2}^{\left(1\right)} & =\hbar \lambda \left(\hat{a}{\hat{\sigma }}_{+}e^{-i\omega_{c}t}+{\hat{a}}^{†}{\hat{\sigma }}_{-}e^{i\omega_{c}t}\right). \end{array}$$

The Hamiltonian ${\hat{H}}_{1}^{\left(1\right)}$ is a linear combination of operators that form a Lie algebra, then we can apply the Wei-Norman Theorem and write the corresponding time-evolution operator as a product of exponentials [35]

(11) $${\hat{U}}_{1}^{\left(1\right)}=e^{\alpha_{z}{\hat{\sigma }}_{z}}e^{\alpha_{+}{\hat{\sigma }}_{+}}e^{\alpha_{-}{\hat{\sigma }}_{-}}.$$

While for ${\hat{U}}_{2}^{\left(1\right)}$ we have the equation:

(12) $$i\hbar \frac{\partial {\hat{U}}_{2}^{\left(1\right)}}{\partial t}=\left[{\hat{U}}_{1}^{(1)†}{\hat{H}}_{2}^{\left(1\right)}{\hat{U}}_{1}^{\left(1\right)}\right]{\hat{U}}_{2}^{\left(1\right)},$$

transforming the interaction we get:

(13) $$\left[{\hat{U}}_{1}^{(1)†}{\hat{H}}_{2}^{\left(1\right)}{\hat{U}}_{1}^{\left(1\right)}\right]≃\hbar \lambda \left(\hat{a}{\hat{\sigma }}_{+}e^{-i\omega_{c}t-2\alpha_{z}}+{\hat{a}}^{†}{\hat{\sigma }}_{-}e^{i\omega_{c}t+2\alpha_{z}}\right),$$

where we have used the fact that the atom-field coupling $\lambda \ll \omega_{a}$ and $\omega_{a}≃\omega_{c}$ so that the RWA is appropriate. Notice that this interaction Hamiltonian has a similar structure as that of Jaynes-Cummings (JC) interaction, and it is important to highlight that the total number of excitations remains constant under this interaction.

Substituting Equation (11) into Schrödinger’s equation we obtain the following set of ordinary, nonlinear, coupled differential equations for the functions $\alpha_{i}$

(14) $$\begin{array}{cc} {\dot{\alpha }}_{z} & =-i\left(\frac{\omega_{a}}{2}-2\lambda \beta^{*}e^{2\alpha_{z}+i\omega_{c}t}\alpha_{+}\right),\\ {\dot{\alpha }}_{+} & =-i\lambda \left(\beta e^{-2\alpha_{z}-i\omega_{c}t}+2\beta^{*}\alpha_{+}^{2}e^{2\alpha_{z}+i\omega_{c}t}\right),\\ {\dot{\alpha }}_{-} & =-i\lambda \beta^{*}e^{2\alpha_{z}+i\omega_{c}t}, \end{array}$$

which we solved with Mathematica. Now we introduce the operators [9,49,50]:

(15) $$\hat{c}=\frac{1}{\sqrt{\hat{M}}}\hat{a}{\hat{\sigma }}_{+},\;\;\;\;{\hat{c}}^{†}={\hat{a}}^{†}{\hat{\sigma }}_{-}\frac{1}{\sqrt{\hat{M}}},$$

with $\hat{M}=\hat{n}+\frac{1}{2}\left(1+{\hat{\sigma }}_{z}\right)$ the total number of excitations in a given ladder. The basis states for the JC Hamiltonian are {|n,e〉,|n+1,g〉} (M=n+1) corresponding to a state where the atom is in its excited state and the field has *n* photons and a state where the atom is in its ground state and the field has n+1 photons. The state |0,g〉 (M=0) where the atom is in its ground state and the field in the vacuum state does not couple with any state. The action of these operators upon the basis states is:

(16) $$\begin{array}{cc} \hat{c}|n,e\rangle & =0,\;\hat{c}|n+1,g\rangle =|n,e\rangle ,\\ {\hat{c}}^{†}|n,e\rangle & =|n+1,g\rangle ,\;{\hat{c}}^{†}|n+1,g\rangle =0,\\ \hat{M}|n,e\rangle & =\left(n+1\right)|n,e\rangle ,\;\hat{M}|n+1,g\rangle =\left(n+1\right)|n+1,g\rangle . \end{array}$$

From the above expressions we obtain the commutation relations

(17) $$\left[\hat{c},{\hat{c}}^{†}\right]={\hat{\sigma }}_{z},\;\;\;\;\left[{\hat{\sigma }}_{z},\hat{c}\right]=2\hat{c},\;\;\;\;\left[{\hat{\sigma }}_{z},{\hat{c}}^{†}\right]=-2{\hat{c}}^{†},$$

and ${\hat{c}}^{2}$, ${\hat{c}}^{†2}$ acting upon any basis state is zero. The interaction Hamiltonian can be written in terms of the operators $\hat{c}$, ${\hat{c}}^{†}$ as:

(18) $$\left[{\hat{U}}_{1}^{(1)†}{\hat{H}}_{2}^{\left(1\right)}{\hat{U}}_{1}^{\left(1\right)}\right]=\hbar \lambda \sqrt{n+1}\left(\hat{c}e^{-i\omega_{c}t-2\alpha_{z}}+{\hat{c}}^{†}e^{i\omega_{c}t+2\alpha_{z}}\right),$$

then we have

(19) $$i\hbar \frac{\partial {\hat{U}}_{2}^{\left(1\right)}}{\partial t}=\hbar \lambda \sqrt{n+1}\left(\hat{c}e^{-i\omega_{c}t-2\alpha_{z}}+{\hat{c}}^{†}e^{i\omega_{c}t+2\alpha_{z}}\right){\hat{U}}_{2}^{\left(1\right)},$$

whose solution has the form (invoking again the Wei-Norman Theorem)

(20) $${\hat{U}}_{2}^{\left(1\right)}=e^{\epsilon_{1}{\hat{c}}^{†}}e^{\epsilon_{2}\hat{c}}e^{\epsilon_{3}{\hat{\sigma }}_{z}},$$

with complex, time dependent functions $\epsilon_{i}$ such that

(21) $$\begin{array}{cc} {\dot{\epsilon }}_{1} & =-i\lambda \sqrt{n+1}\left(e^{i\omega_{c}t+2\alpha_{z}}-\epsilon_{1}^{2}e^{-i\omega_{c}t-2\alpha_{z}}\right),\\ {\dot{\epsilon }}_{2} & =-i\lambda \sqrt{n+1}\left(1+2\epsilon_{1}\epsilon_{2}\right)e^{-i\omega_{c}t-2\alpha_{z}},\\ {\dot{\epsilon }}_{3} & =-i\lambda \sqrt{n+1}\epsilon_{1}e^{-i\omega_{c}t-2\alpha_{z}}, \end{array}$$

and with the initial condition $\epsilon_{1}\left(0\right)=\epsilon_{2}\left(0\right)=\epsilon_{3}\left(0\right)=0$.

Finally, taking into account the above relationships and in particular (4), (11) and (20), the full time evolution operator for the hybrid system is:

(22) $$\hat{U}\left(t\right)={\hat{U}}_{0}\left(t\right){\hat{U}}_{1}^{\left(1\right)}\left(t\right){\hat{U}}_{2}^{\left(1\right)}\left(t\right),$$

where each term has been written as a product of exponentials and can be applied easily to any given initial state so that the construction of the evolved wavefunction is relatively straightforward. This result is our main contribution. It is valid whenever the coupling between the field and the mechanical oscillator satisfies $G/\omega_{m}\ll 1$ and when the atom-field coupling satisfies $\lambda /\omega_{a}\ll 1$. With the set of parameters we used in this work, namely $G/\omega_{c}=3\times 10^{-4}$, $\lambda /\omega_{c}=1.25\times 10^{-2}$ we can expect that the evolution operator can be safely applied to propagate the system up to times of the order of $10^{4}T_{c}$ with $T_{c}=2\pi /\omega_{c}$. It is a major challenge to obtain analytic expressions for the evolution of forced optomechanical systems even when the system is not an open quantum system.

## 3. Evaluation of Observables

Let us consider an initial state given by |Ψ(0)〉=|n〉⊗|e〉⊗|Γ〉 corresponding to cavity with *n* photons, a two level atom in its excited state and a mechanical oscillator in a coherent state Γ. Applying the operator ${\hat{U}}_{I}^{\left(1\right)}={\hat{U}}_{1}^{\left(1\right)}{\hat{U}}_{2}^{\left(1\right)}$ to the initial state we get:

(23) $$\begin{array}{cc} {\hat{U}}_{2}^{\left(1\right)}|n,e\rangle ⊗|\Gamma \rangle = & e^{\epsilon_{3}}\left[|n,e\rangle +\epsilon_{1}|n+1,g\rangle \right]⊗|\Gamma \rangle ,\\ {\hat{U}}_{1}^{\left(1\right)}\left[{\hat{U}}_{2}^{\left(1\right)}|n,e\rangle ⊗|\Gamma \rangle \right]= & e^{\epsilon_{3}}\left[e^{\alpha_{z}}\left(1+\alpha_{+}\alpha_{-}\right)|n,e\rangle +\alpha_{-}e^{-\alpha_{z}}|n,g\rangle \right]⊗|\Gamma \rangle \\ & +e^{\epsilon_{3}}\epsilon_{1}\left[e^{-\alpha_{z}}|n+1,g\rangle +\alpha_{+}e^{\alpha_{z}}|n+1,e\rangle \right]⊗|\Gamma \rangle , \end{array}$$

due to the forcing term in the Hamiltonian the total number of excitations is no longer constant; in contrast with the JC Hamiltonian. This state can be written as:

(24) $$\begin{array}{cc} {\hat{U}}_{I}^{\left(1\right)}|n,e\rangle ⊗|\Gamma \rangle = & \left[c_{1}\left(t\right)|n,e\rangle +c_{2}\left(t\right)|n,g\rangle +c_{3}\left(t\right)|n+1,g\rangle +c_{4}\left(t\right)|n+1,e\rangle \right]⊗|\Gamma \rangle . \end{array}$$

If instead of a number state for the field we have a coherent state |α〉, we get

(25) $$\begin{array}{cc} {|\Psi \left(t\right)\rangle }_{I}= & {\hat{U}}_{I}^{\left(1\right)}|\alpha ,e\rangle ⊗|\Gamma \rangle ,\\ = & \sum_{n=0}^{\infty }c_{n}\left[c_{1}\left(t\right)|n,e\rangle +c_{2}\left(t\right)|n,g\rangle +c_{3}\left(t\right)|n+1,g\rangle +c_{4}\left(t\right)|n+1,e\rangle \right]⊗|\Gamma \rangle , \end{array}$$

where $c_{n}=\exp[-\frac{1}{2}{\left|\alpha \right|}^{2}]\alpha^{n}/\sqrt{n!}$. The time evolution operator ${\hat{U}}_{0}$ does not involve the atomic degrees of freedom, then we can use Equation (25) to evaluate the atomic evolution. For instance, the probability to find the atom in its excited state at time *t* is given by

(26) $$P_{e}\left(\alpha ,t\right)={\left|{\langle e|\Psi \left(t\right)\rangle }_{I}\right|}^{2}={\left|\sum_{n=0}^{\infty }c_{n}\left[c_{1}\left(t\right)|n\rangle +c_{4}\left(t\right)|n+1\rangle \right]\right|}^{2},$$

**Figure 1:**
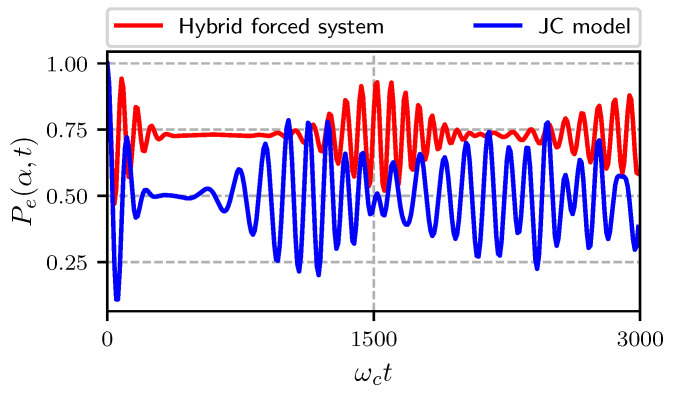
Probability to find the atom in its excited state $P_{e}\left(\alpha ,t\right)$ with α=2, $\omega_{a}/\omega_{c}=0.95$, $\omega_{L}/\omega_{c}=0.5$, $\lambda /\omega_{c}=0.0125$, $\Omega /\omega_{c}=0.01$. In red we show the case for a forced system, in blue the Jaynes-Cummings model (JCM) result.

In Figure 1 we show the probability for the atom to be in its excited state for the hybrid pumped system (red) and for the JC Hmiltonian (blue). The initial state of the cavity is a coherent state with an average number of photons $\bar{n}=4$ and atom-cavity coupling constant $\lambda /\omega_{c}=0.0125$, the pumping amplitude is $\Omega /\omega_{c}=0.01$ and the atomic frequency is $\omega_{a}/\omega_{c}=0.95$. In both cases we can see the usual pattern of quantum collapse and revivals present in the JCM, however the length of the collapse and the definition of the revivals is not the same. In the hybrid pumped case, the time between the collapse and the first revival is longer than in the JC case; the definition of the revival is more definite in the pumped case than in the JC case and the probability to find the atom in its excited state is larger for the pumped case.

Let us consider now the average value of the photon number operator; it is given by:

(27) $$\begin{array}{cc} \langle \hat{n}\left(t\right)\rangle & =\langle \Psi \left(t_{0}\right)|{\hat{U}}_{I}^{(1)†}{\hat{U}}_{0}^{†}\hat{n}{\hat{U}}_{0}{\hat{U}}_{I}^{\left(1\right)}|\Psi \left(t_{0}\right)\rangle \\ \; & ={\;}_{I}\langle \Psi \left(t\right)|{\hat{n}}_{I}\left(t\right){|\Psi \left(t\right)\rangle }_{I}, \end{array}$$

with ${\hat{n}}_{I}\left(t\right)$ the photon number operator in the interaction picture. Taking the explicit form of the operator ${\hat{U}}_{0}$ (see Equation (4)) we obtain

(28) $${\hat{n}}_{I}\left(t\right)=\hat{n}+\beta^{*}\hat{a}+\beta {\hat{a}}^{†}+{\left|\beta \right|}^{2},$$

and ${|\Psi \left(t\right)\rangle }_{I}$ given by Equation (25). For the phonon number operator we get

(29) $${\hat{N}}_{I}\left(t\right)=\hat{N}+\left(\alpha_{4}{\hat{b}}^{†}+\alpha_{4}^{*}\hat{b}\right){\hat{n}}_{I}\left(t\right)+{\left|\alpha_{4}\right|}^{2}{\hat{n}}_{I}^{2}\left(t\right),$$

and we see that the phonon number operator depends on the number of photons present in the cavity. Since the pumping term modifies the photon number, then it will also modify the phonon number evolution. We can now evaluate observables like the photon and phonon dispersion. We present our numerical results in the following section.

## 4. Numerical Results, Unitary Evolution

**Figure 2:**
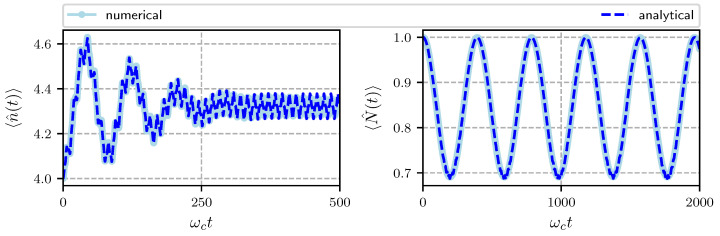
Temporal evolution of the average photon number (**left**) and temporal evolution of the average phonon number (**right**). Analytical results in dark-blue, numerical results in light-blue. Hamiltonian parameters α=2, $\omega_{a}/\omega_{c}=0.95$, $\omega_{L}/\omega_{c}=0.5$, $\omega_{m}/\omega_{c}=0.016$, $G/\omega_{c}=0.00032$, $\lambda /\omega_{c}=0.0125$, $\Omega /\omega_{c}=0.01$.

**Figure 3:**
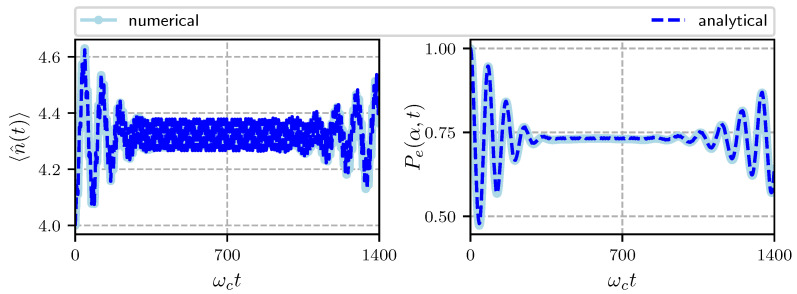
Temporal evolution of the average photon number (**left**) and probability to find the atom in its excited state (**right**) with Hamiltonian parameters α=2, $\omega_{a}/\omega_{c}=0.95$, $\omega_{L}/\omega_{c}=0.5$, $\omega_{m}/\omega_{c}=0.016$, $G/\omega_{c}=0.00032$, $\lambda /\omega_{c}=0.0125$, $\Omega /\omega_{c}=0.01$. We present numerical (light-blue) and analytical (dark-blue) calculations

In order to test the validity of our approximations, we also made a purely numerical calculation of the average value of the photon, phonon number operators and the Wigner function using Python [51]. In Figure 2 we show the numerical and the analytical results for the temporal evolution of the average photon number and the average phonon number for Hamiltonian parameters specified in the caption. The initial state of the system is |Ψ(0)〉=|α〉⊗|Γ〉⊗|e〉 corresponding to an atom in its excited state, the cavity field in a coherent state with α=2 and the mechanical oscillator in a coherent state with Γ=1. The average phonon number oscillates with frequency $\omega_{m}$ from its initial value of one to approximately 0.7 so that the mechanical oscillator is cool down. This can be understood since photons impinging at a frequency red-detuned from the cavity resonance will preferentially scatter upward in energy in order to enter the cavity resonance, absorbing a phonon from the oscillator. On the other hand, the average photon number shows much faster oscillations with frequency $\omega_{c}$ which are due to the exchange between the cavity field and the two-level atom, we can see at longer times the quantum collapse and revivals familiar with the Jaynes-Cummings model (see also Figure 3 right panel).

The evolution is done for the interval $0\leq \omega_{c}t\leq 500$ for the photons and $0\leq \omega_{c}t\leq 2000$ for the phonons. We can see an excellent agreement between the analytic and the numerical calculations. For the photons we used an initial coherent state with α=2 and for the phonons a coherent state with Γ=1. Notice that the pumping frequency is far from the resonance cavity frequency $\omega_{c}$.

In Figure 3 we show the temporal evolution of the photon number operator with initial condition α=2 corresponding to $\langle \hat{n}\rangle ={\left|\alpha \right|}^{2}=4$ (left) and the probability to find the atom in its excited state (right). We see an exchange of excitations between the atom and the field, at the beginning of the evolution the probability for the atom to remain in its excited state $P_{e}\left(\alpha ,t\right)$ decreases to about 0.5 and at the same time the average number of photons increases to about 4.5, notice also the rapid oscillations with small amplitude around an average value for the number operator, these are due to the forcing term. After some time, $P_{e}\left(\alpha ,t\right)$ attains a constant value around 0.75 and the average photon number oscillates around 4.3 until $\omega_{c}t≃1000$ where the first revival occurs. The overall behavior of the average photon number can be guessed from $P_{e}\left(\alpha ,t\right)$.

**Figure 4:**
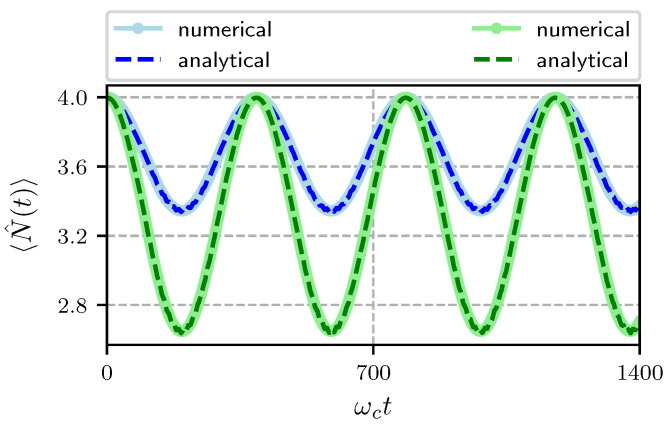
Temporal evolution of the average phonon number with Hamiltonian parameters $\omega_{a}/\omega_{c}=0.95$, $\omega_{L}/\omega_{c}=0.9$, $\omega_{m}/\omega_{c}=0.016$, $G/\omega_{c}=0.00032$, $\lambda /\omega_{c}=0.0125$, $\Omega /\omega_{c}=0.01$ and $\left\{\alpha ,\Gamma \right\}=\left\{2,2\right\}$ (blue), and $\left\{\alpha ,\Gamma \right\}=\left\{3,2\right\}$ (green).

In Figure 4 we show the temporal evolution of the average phonon number for different amplitudes of the cavity field and Hamiltonian parameters given in the caption. The initial state of the atom is the excited state. In blue we show the case when the initial state of the field is a coherent state with α=2 and $\langle \hat{n}\left(0\right)\rangle ={\left|\alpha \right|}^{2}=4$ and in green we plot the case when the initial state of the field is a coherent state with α=3, $\langle \hat{n}\left(0\right)\rangle =9$. In both cases the initial state of the mechanical oscillator is a coherent state with Γ=2, $\langle \hat{N}\left(0\right)\rangle =4$. We have used a pump frequency near resonance $\omega_{L}=0.9\omega_{c}$. As mentioned before, since we are dealing with red detuning we expect power flow from the mechanical mode to the optical mode [52]. We see that 〈N(t)〉 evolves periodically with the frequency of the mechanical oscillator, it decreases from its initial value and after a period it returns to it. We see from Equation (29) that the average phonon number depends upon the function $\alpha_{4}$ and the average photon number. The former is a function of the coupling constant *G* and the frequency of the mechanical oscillator, the later is a function of the field’s frequency through the function β (see Appendix A).

Notice that the decrease is larger for the case when the average number of photons is larger so that one can manipulate the number of phonons by means of the interaction time, the amplitude of the cavity field and the frequency of the forcing term. We also show in this plot the results obtained with a purely numerical calculation and we can see a very good agreement between them.

**Figure 5:**
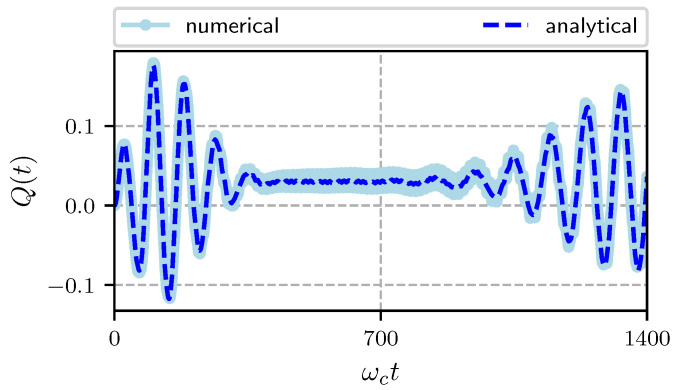
Temporal evolution of the Mandel parameter Q(t) with Hamiltonian parameters $\omega_{a}/\omega_{c}=0.95$, $\omega_{L}/\omega_{c}=0.5$, $\omega_{m}/\omega_{c}=0.016$, $G/\omega_{c}=0.00032$, $\lambda /\omega_{c}=0.0125$, $\Omega /\omega_{c}=0.01$ and α=2.

**Figure 6:**
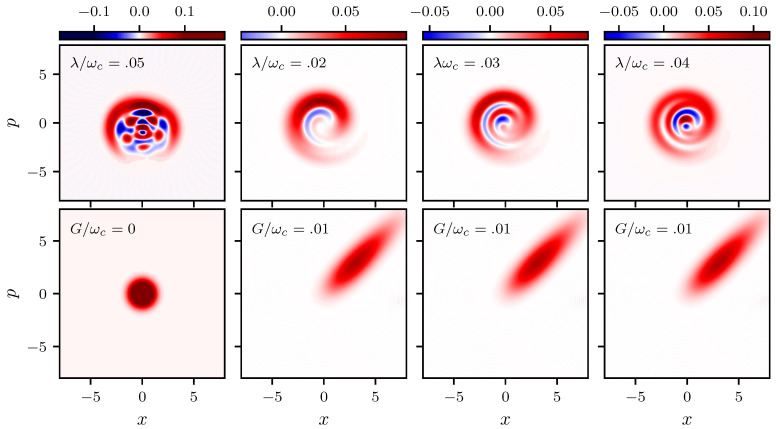
Plot of the Wigner function for a field coherent state (**first row**) and for a vacuum state of the mechanical oscillator (**second row**) both evaluated at time $\omega_{c}t=100$.

We now present the temporal evolution of the Mandel *Q* parameter defined as:

(30) $$Q\left(t\right)=\frac{\langle {\hat{n}}^{2}\left(t\right)\rangle -{\langle \hat{n}\left(t\right)\rangle }^{2}}{\langle \hat{n}\left(t\right)\rangle }-1,$$

for a state with *Q* in the range −1≤Q<0 the statistics is sub-Poissonian, and if Q>0, super-Poissonian. For a coherent state Q=0. In Figure 5 we plot the temporal evolution of the *Q* function for an initial coherent state |α〉 with |α|=2. It starts at zero as corresponds to a coherent state, as time evolves it oscillates around zero alternating between positive and negative values, that is between super and sub-Poissonian statistics this happens in the same temporal region where the exchange of excitations between the field and the atom is most important. After some time it oscillates above zero with a small amplitude (when the probability to find the atom in its excited state is constant) and remains with a super-Poissonian statistics until the revival time (see Figure 3) when the oscillations around zero repeat themselves. Finally, to glimpse the non-classical features of the system the Wigner function plays an important role. In Figure 6 we plot the Wigner function for both the field and the mechanical oscillator at time $\omega_{c}t=100.$ In the figure we vary the strength of the atom-cavity coupling λ and keep fixed the coupling between the mechanical oscillator and the cavity field *G* as specified in the figure. In the left column we present the Wigner function evaluated at time $\omega_{c}t=100$ for a Jaynes-Cummings model ($G/\omega_{c}=0$) and $\lambda /\omega_{c}=0.05$, it shows the generation of multi-component Schrödinger cats; we found that for $\lambda /\omega_{c}\leq 0.02$ distribution is a Gaussian function and for $\lambda /\omega_{c}>0.02$ it displays the non classical behavior corresponding to a multi component Schrödinger cat. We see in the figure that for the hybrid system ($G/\omega_{c}\neq 0$) the behavior of the Wigner function differs significantly. The coupling between the mechanical oscillator and the cavity field is so relevant that even for a small value of $G/\omega_{c}$ the distribution is smeared in a ring. For larger values of $\lambda /\omega_{c}$ and the same value of $G/\omega_{c}$, we see that the Wigner function for the field takes negative values (shown in blue) and the non classicality of the system increases with the strength of the coupling $\lambda /\omega_{c}$. The Wigner function for the pumped optomechanical system shows squeezing and a small negativity independently of $\lambda /\omega_{c}$.

## 5. Conclusions

In this work we have presented an approximate method to construct the time evolution operator for a hybrid system composed of a forced optomechamical system and a two-level atom inside the cavity, the atom interacts only with the cavity field by means of a Jaynes-Cummings interaction. In order to solve the problem we split the Hamiltonian as the sum of a forced optomechanical Hamiltonian and that of the free atom with the JC interaction. The time evolution operator for the forced optomechanical Hamiltonian is approximated as a product of exponentials [42] and it is then used to take the JC interaction into a generalized interaction picture. As a result we obtained cumbersome expressions for the transformed operators which we approximated by neglecting terms of the order $G/\omega_{m}$ and ${\left(G/\omega_{m}\right)}^{2}$ as compared to one. Within this approximation the interaction picture Hamiltonian becomes that of a free two level atom and a displaced JC interaction whose exact time evolution operator we constructed using the Wei-Norman Theorem. Once we have the full time evolution operator we can obtain the average value of any observable, as an example we evaluated the temporal evolution of the average photon and phonon number operators, the probability to find the atom in its excited state, the Mandel parameter for the cavity field and the Wigner function. We used as initial state |Ψ(0)〉=|α〉⊗|e〉⊗|Γ〉 where |α〉 is the ket corresponding to the cavity field in a coherent state α, |e〉 is that corresponding to the atom in its excited state and |Γ〉 is the ket for the mechanical oscillator in a coherent state Γ. The average number of photons is a function of the pumping amplitude Ω and the pump frequency $\omega_{L}$, when $\omega_{L}≃\omega_{c}$ there is a periodic growth in the number of photons and the amplitude of this growth is proportional to Ω. The average number of phonons is a periodic function of time which depends also on the optomechanical coupling $G/\omega_{m}$ and on the number of photons present in the cavity. For red detuning there is a power flow from the mechanical mode to the optical mode and the cooling of the mechanical mode is more important as the number of photons increases. Since the evolution of the phonon number is periodic one can select an interaction time such that the number of phonons be at a minimum. We also evaluated the Mandel parameter for the cavity field and we found that it alternates between sub-Poissonian and super-Poissonian statistics in the region of time where there is an important exchange of excitations between the atom and the cavity field. We stress the fact that our approximations are done in the interaction Hamiltonian where we have neglected terms proportional to $G/\omega_{m}$ and ${\left(G/\omega_{m}\right)}^{2}$ with respect to one. The excellent agreement between the analytic and the numerical results obtained using the full Hamiltonian as given in (1) indicate the validity of our approximations.

## Appendix A. The Wei–Norman Approach

Here we describe the method we used to obtain the time evolution operator for the forced optomechanical system. The first thing to notice is that the set of operators appearing in ${\hat{H}}_{o}$ is closed under commutation

	$\hat{n}$	$\hat{N}$	$\hat{n}\hat{b}$	$\hat{n}{\hat{b}}^{†}$	${\hat{n}}^{2}$
$\hat{n}$	0	0	0	0	0
$\hat{N}$	0	0	$-\hat{n}\hat{b}$	$\hat{n}{\hat{b}}^{†}$	0
$\hat{n}\hat{b}$	0	$\hat{n}\hat{b}$	0	${\hat{n}}^{2}$	0
$\hat{n}{\hat{b}}^{†}$	0	$-\hat{n}{\hat{b}}^{†}$	$-{\hat{n}}^{2}$	0	0
${\hat{n}}^{2}$	0	0	0	0	0

In this table, we had to incorporate the operator ${\hat{n}}^{2}$ that arises from the commutator between $\hat{n}\hat{b}$ and $\hat{n}{\hat{b}}^{†}$. The time evolution operator corresponding to the Hamiltonian ${\hat{H}}_{o}$ can then be written *exactly* as a product of exponentials [35,46],

(A1) $${\hat{U}}_{o}\left(t\right)=e^{\alpha_{1}\hat{n}}e^{\alpha_{2}\hat{N}}e^{(\alpha_{3}+|\alpha_{4}{|}^{2}/2){\hat{n}}^{2}}{\hat{D}}_{\hat{b}}\left(\alpha_{4}\hat{n}\right).$$

The time-dependent functions $\alpha_{i}$ are obtained after substitution of Equation (A1) into Schrödinger’s equation. As a result we get:

(A2) $$\begin{array}{cc} \alpha_{1} & =-i\omega_{c}t,\\ \alpha_{2} & =-i\omega_{m}t,\\ \alpha_{3} & =-{\left(\frac{G}{\omega_{m}}\right)}^{2}\left[-i\omega_{m}t+\left(1-e^{-i\omega_{m}t}\right)\right],\\ \alpha_{4} & =-\frac{G}{\omega_{m}}\left(1-e^{i\omega_{m}t}\right). \end{array}$$

Once we know the exact time evolution operator for the optomechanical system, we transform the forcing term to obtain an interaction picture Hamiltonian

(A3) $${\hat{H}}_{I}^{\left(0\right)}=\hbar \Omega \cos\left(\omega_{L}t\right)\left[{\hat{U}}_{o}{\left(t\right)}^{†}\left(\hat{a}+{\hat{a}}^{†}\right){\hat{U}}_{o}\left(t\right)\right],$$

applying the transformation we obtain:

(A4) $$\begin{array}{cc} {\hat{U}}_{o}^{†}\hat{a}{\hat{U}}_{o} & =e^{iE\left(t\right)\left(2\hat{n}+1\right)}e^{iF\left(t\right)\left[{\hat{b}}^{†}e^{i\frac{\omega_{m}}{2}t}+\hat{b}e^{-i\frac{\omega_{m}}{2}t}\right]}\hat{a}e^{-i\omega_{c}t},\\ {\hat{U}}_{o}^{†}{\hat{a}}^{†}{\hat{U}}_{o} & ={\hat{a}}^{†}e^{i\omega_{c}t}e^{-iF\left(t\right)\left[{\hat{b}}^{†}e^{i\frac{\omega_{m}}{2}t}+\hat{b}e^{-i\frac{\omega_{m}}{2}t}\right]}e^{-iE\left(t\right)\left(2\hat{n}+1\right)}, \end{array}$$

where

(A5) $$\begin{array}{cc} F(t) & =2\left(\frac{G}{\omega_{m}}\right)\sin\left(\frac{\omega_{m}}{2}t\right),\\ E(t) & ={\left(\frac{G}{\omega_{m}}\right)}^{2}\left(\omega_{m}t-\sin\left(\omega_{m}t\right)\right). \end{array}$$

Notice the presence of the operators in the exponentials. These impede the application of the Wei-Norman theorem since the set of operators is not closed under commutation. Notice however that the factor $G/\omega_{m}\ll 1$ [36] so that at this point it is convenient to make the approximation

(A6) $${\hat{U}}_{opt}^{†}\hat{a}{\hat{U}}_{opt}≃\hat{a}e^{-i\omega_{c}t},\;\;\;{\hat{U}}_{opt}^{†}{\hat{a}}^{†}{\hat{U}}_{opt}≃{\hat{a}}^{†}e^{i\omega_{c}t},$$

and we get the approximate interaction Hamiltonian

(A7) $${\tilde{H}}_{I}^{\left(0\right)}=\hbar \Omega \cos\left(\omega_{L}t\right)\left(\hat{a}e^{-i\omega_{c}t}+{\hat{a}}^{†}e^{i\omega_{c}t}\right),$$

where we have used ${\tilde{H}}_{I}^{\left(0\right)}$ to stress the fact that it is an approximate interaction Hamiltonian. The corresponding exact time evolution operator can be written as a product of exponentials

(A8) $${\hat{U}}_{I}^{\left(0\right)}=e^{\beta {\hat{a}}^{†}}e^{\gamma \hat{a}}e^{\delta },$$

with:

(A9) $$\begin{array}{cc} \dot{\beta } & =-i\Omega \cos\left(\omega_{L}t\right)e^{i\omega_{c}t},\\ \dot{\gamma } & =-i\Omega \cos\left(\omega_{L}t\right)e^{-i\omega_{c}t},\\ \dot{\delta } & =\beta \dot{\gamma }, \end{array}$$

and initial conditions β(0)=γ(0)=δ(0)=0. We see that $\beta =-\gamma^{*}$ so that we can write

(A10) $$\begin{array}{cc} {\hat{U}}_{I}^{\left(0\right)} & =e^{\delta }e^{\frac{1}{2}{\left|\beta \right|}^{2}}e^{\beta {\hat{a}}^{†}-\beta^{*}\hat{a}}\\ \; & =e^{\delta +\frac{1}{2}{\left|\beta \right|}^{2}}{\hat{D}}_{\hat{a}}\left(\beta \right). \end{array}$$

Finally, taking into account the above relationships and Equation (2), the approximate evolution operator of the forced optomechanical system is [42]

(A11) $$\begin{array}{cc} {\hat{U}}_{0} & ={\hat{U}}_{o}{\hat{U}}_{I}^{\left(0\right)}\\ \; & =e^{\delta +\frac{1}{2}{\left|\beta \right|}^{2}}e^{\alpha_{1}\hat{n}}e^{\alpha_{2}\hat{N}}e^{(\alpha_{3}+|\alpha_{4}{|}^{2}/2){\hat{n}}^{2}}{\hat{D}}_{\hat{b}}\left(\alpha_{4}\hat{n}\right){\hat{D}}_{\hat{a}}\left(\beta \right). \end{array}$$
